# What's in a Day? A Guide to Decomposing the Variance in Intensive Longitudinal Data

**DOI:** 10.3389/fpsyg.2016.00891

**Published:** 2016-06-14

**Authors:** Silvia de Haan-Rietdijk, Peter Kuppens, Ellen L. Hamaker

**Affiliations:** ^1^Methodology and Statistics, Faculty of Social and Behavioural Sciences, Utrecht UniversityUtrecht, Netherlands; ^2^Department of Psychology, Faculty of Psychology and Educational Sciences, Katholieke Universiteit LeuvenLeuven, Belgium

**Keywords:** intensive longitudinal data, experience sampling, multilevel analysis, dynamical modeling, autoregression, emotional inertia, variance decomposition, code:R

## Abstract

In recent years there has been a growing interest in the use of intensive longitudinal research designs to study within-person processes. Examples are studies that use experience sampling data and autoregressive modeling to investigate emotion dynamics and between-person differences therein. Such designs often involve multiple measurements per day and multiple days per person, and it is not clear how this nesting of the data should be accounted for: That is, should such data be considered as two-level data (which is common practice at this point), with occasions nested in persons, or as three-level data with beeps nested in days which are nested in persons. We show that a significance test of the day-level variance in an empty three-level model is not reliable when there is autocorrelation. Furthermore, we show that misspecifying the number of levels can lead to spurious or misleading findings, such as inflated variance or autoregression estimates. Throughout the paper we present instructions and R code for the implementation of the proposed models, which includes a novel three-level AR(1) model that estimates moment-to-moment inertia and day-to-day inertia. Based on our simulations we recommend model selection using autoregressive multilevel models in combination with the AIC. We illustrate this method using empirical emotion data from two independent samples, and discuss the implications and the relevance of the existence of a day level for the field.

In psychological research it is increasingly common to study the dynamics of within-person processes by collecting intensive longitudinal data (ILD; cf. Walls and Schafer, 2006), that is, many repeated measurements for multiple persons. Examples of ILD research designs include diary reports, observational methods, and experience sampling methods (ESM, also referred to as ambulatory assessment; cf. Trull and Ebner-Priemer, 2013) which have become highly feasible and efficient thanks to the widespread use of devices like tablets and smartphones. With the resulting ILD, researchers can analyze each person's time series separately, or use a multilevel model to account for the nesting of measurements in persons and to study interpersonal variation in dynamics.

In ESM and related ILD designs there are often multiple measurements per day, and multiple days per participant. While many researchers have implemented two-level modeling approaches to account for the nesting of measurements within persons and to study interpersonal differences, we can ask whether the day should be treated as an intermediate level, so that measurements in the model are nested within days, and days are nested within persons. Indeed, some researchers have used three-level models to account explicitly for this structure of the data (e.g., van Eck et al., 1998; Peeters et al., 2006; Moberly and Watkins, 2008; Doane and Adam, 2010; Mor et al., 2010; Nisenbaum et al., 2010). In this paper we argue that it is crucial to consider the number of levels in ILD to avoid spurious findings or misleading estimates.

The question whether a day level should be included in the model, i.e., whether there is variation over days, may seem relatively straightforward to answer: By comparing an empty model that consists of two levels (measurements nested within persons) to a three-level empty model (beeps nested in days nested in persons), we should be able to tackle this issue. However, as we will show in this paper, the issue is more complicated when there is autocorrelation in the data. The presence of autocorrelation is to be expected in ILD, and it can lead to the *appearance* of substantial variance at the day level, which results in overfitting if this variance is taken as an indication that three levels are needed. Underfitting, where the day level is omitted from the model even though there is substantial variance at this level, can also be problematic, resulting in inflated or spurious autocorrelation. In both scenarios, researchers run the risk of drawing misleading conclusions based on their estimated models. Therefore, it is important for researchers working with nested longitudinal measurements to explicitly consider how many levels are appropriate before interpreting a specific model. This advice applies also if researchers consider the autocorrelation itself to be of little substantive interest.

The purpose of this article is twofold. First, we wanted to investigate the issue of the day level and the question of how to choose the number of levels when modeling nested ILD. We propose a novel three-level model that can be used to investigate new hypotheses about within-person dynamics. Second, this paper provides a tutorial for multilevel autoregressive (AR) modeling of ILD using R, in which we demonstrate how one can specify meaningful two-level and three-level AR(1) models,^1^ and how one can determine whether two-level or three-level modeling is more appropriate. The implementation of multilevel AR(1) models requires close attention to details, such as how to create the lagged predictor(s), how to center them, how to deal correctly with missing values and with the interruptions caused by the nighttime, and how to make a valid comparison between different models, given that they necessarily have different numbers of usable observations. For this reason, throughout the paper we present instructions and R code for the implementation of the proposed techniques.

This paper is structured as follows. First, we discuss the two-level and three-level AR(1) models and illustrate their implementation in R, using empirical emotion data from two independent samples. The empirical analyses serve a dual function, as they address substantive research questions about the regulation of affect, in addition to illustrating the application of the models. Then, in the section after that, we explore why the significance test of the day-level variance should not be used to choose the number of levels, and why underfitting and overfitting can both occur and lead to problems. In the fourth section we use simulations to evaluate model selection as an alternative approach for deciding on the number of levels. Then, in the fifth section we revisit the empirical data, and lastly we end the paper by summarizing our findings and discussing opportunities for further research.

## 1. The models

In this section we will show how we can specify meaningful two-level and three-level AR(1) models for ILD and how we can implement them in R. First, we provide some background regarding AR(1) modeling of ILD, and regarding the substantive questions that are addressed by our empirical applications. Then we introduce the empirical data, and discuss the software and some necessary preparations required for the analysis, after which we are ready to present and apply the various models.

### 1.1. Background

AR(1) models have previously been applied to ILD to study the regulation of affect and the between-person differences in affect dynamics. In this context, the autoregressive parameter of the AR(1) model is interpreted as the *inertia* of affect, indicating how much carry-over there is from one measurement to the next. This parameter takes an absolute value of one or smaller, with larger values indicating more carry-over, considered to be indicative of weaker affect regulation. Previous research has linked inertia with other person characteristics such as trait neuroticism, depression, and low self-esteem (Suls et al., 1998; Kuppens et al., 2010). Inertia research has been done with observational data (e.g., Kuppens et al., 2012), daily diary data (e.g., Wang et al., 2012; Brose et al., 2015), and ESM data (e.g., Suls et al., 1998; Koval and Kuppens, 2011). The recently developed *network approach* to psychopathology also involves (vector) autoregressive modeling of ESM data (Borsboom and Cramer, 2013; Bringmann et al., 2013).

In inertia research using diary data, each measurement represents a different day, so it is the carry-over of affect *from day to day* that is studied. In contrast, in designs with multiple measurements per day, the measurements reflect different moments nested within days, so that the focus is on the carry-over of affect from moment to moment *within a day*. This raises the question whether the different studies were investigating one and the same regulation mechanism, or actually distinct regulation mechanisms that operate at different time scales. And if the latter is true, then the next question is how the mechanisms are related: Do people who have more carry-over of affect from day to day also, on average, have more carry-over from moment to moment? To be able to address these questions, we will propose a three-level AR(1) model, combining day-to-day and moment-to-moment autoregression in one model.

### 1.2. Introducing the data

In this article we use empirical affect data from two ESM studies, each giving us two outcome variables. The participants in both studies were students at the University of Leuven. Both studies were approved by the Ethics committee of the Faculty of Psychology and Educational Sciences at KU Leuven and all participants gave written informed consent. The first data set (from Koval et al., 2013) contains negative affect (Neg) and positive affect (Pos) scores for 95 persons, over the course of 7 days (although three participants provided data for an 8th day), with a maximum of 10 measurements on each day. The second data set (from Pe and Kuppens, 2012) contains negative and positive affect scores for 79 persons, over the course of 6–21 days, with 14 days being the average length. As in the first data set, there were at most 10 measurements per day. In both studies, the participants rated several specific positive and negative emotions on a slider scale running from 1 to 100, and the Neg and Pos scores were obtained by averaging over the specific negative and positive emotions, respectively. By using both data sets, we have an opportunity to compare findings for different samples. Since the second study included more days (on average), it can be expected that an analysis using this data set has more statistical power.

### 1.3. Preparations

We implement our analyses with the open-source statistical software R (version 3.1.2; R Core Team, 2014), together with the packages lme4 (version 1.1-7; Bates et al., 2014), lmerTest (version 2.0-11; Kuznetsova et al., 2013), and DataCombine (Gandrud, 2014). Although, it should be possible to fit the models in other multilevel software like HLM (Raudenbush et al., 2004), we use R because it is highly flexible, freely available, and it can be used for all steps of the analysis from preparing the data to summarizing the model results.

For our analyses the data need to have the right format within R. We start out with a dataframe in the R workspace called “ESM,” which contains the data set (for one sample) in long format, that is, with a different row for each measurement occasion. Each column in the data frame represents a different variable, and we have the numeric affect variables “Neg” and “Pos,” in addition to the integer variables “Person,” “Day,” and “Beep,” which together indicate the person (1 to Np) and the occasion for each affect score. For use in our analysis, we create factor variables for person and day, by specifying:


ESM$PersonF <- factor(x=ESM$Person)
ESM$DayF <- factor(x=ESM$Day)


Note that it would be no problem if different persons had different numbers of rows in the dataframe, for example, if in a given study some persons had more days or more measurements on some days than other persons. If there are missing values in between observations, they need to be coded with the value *NA* in R. Importantly, the rows of the dataframe should have a consistent ordering, with the beeps sorted in ascending order within the days, and the days sorted in ascending order within the persons. For instance, if person 1 is measured 10 times a day for 7 days, as in our empirical data, the first 70 rows in the data set should contain the data for this person, with the first 10 rows representing that person's first day of measurement, etcetera.

### 1.4. Two-level models

We start by looking at the two-level modeling approach. First we consider the most basic, empty (or intercepts-only) two-level model, which accounts for the fact that we have measurements within persons, but which does not include an autoregressive parameter. This empty model partitions the variance into variance at the person level and variance at the measurement level, and as we will show, the estimates of this model provide us with the lagged centered predictor that we need for the two-level AR(1) model, which is presented next.

#### 1.4.1. Empty two-level model

The empty two-level model provides insight into the relative magnitude of between-person variability and within-person variability over time. Let *y*_*bi*_ be the affect score of person *i* at measurement (beep) *b*. The model is given by

(1) $$\begin{array}{lll} \text{Level }1: & y_{bi}=\mu_{i}+e_{bi}\; & \; \end{array}$$

(2) $$\begin{array}{lll} \text{Level }2: & \mu_{i}=\gamma_{00}+u_{0i},\; & \; \end{array}$$

where μ_*i*_ represents the mean or trait level of person *i*, *e*_*bi*_ their deviation from this trait level at beep *b*, γ_00_ the grand mean (or average trait level) in the population, and *u*_0*i*_ the deviation of person *i*'s trait level from this grand mean. The three parameters that need to be estimated are the average trait level γ_00_, the person-level variance $\sigma_{u_{0}}^{2}$, and the beep-level variance $\sigma_{e}^{2}$.

We use the lmer() function (after loading the packages lme4 and lmerTest) to estimate the model, by specifying the model equation, the name of the dataframe, and selecting regular Maximum Likelihood or Restricted Maximum Likelihood estimation (we choose the former):


twolevel.empty <- lmer(Neg ~ 1 + (1 |
PersonF),
data = ESM, REML = FALSE)


To extract a summary of the model results from the stored object “twolevel.empty” we specify


summary(twolevel.empty)


Similar code was used for the Neg variable in the other data set, and for the Pos variables in both samples. The estimated parameters for all four outcome variables are given in the top part of Table 1. We report the standard deviations, rather than the variances, because we find the standard deviations easier to interpret and compare. As can be seen in the table, there is substantial variation over persons in the trait levels of negative and positive affect, but the variation in people's state levels over time is a bit larger.

**Table 1:** **The estimated parameters from the two-level empty model and AR(1) model, for negative affect (***Neg***) and positive affect (***Pos***) in the two samples**.

**Two-level empty model**	**Notation**	***Neg1***	***Neg2***	***Pos1***	***Pos2***
Avg. trait level	γ_00_	15.65 (1.10)	11.21 (0.90)	57.26 (1.34)	58.69 (1.40)
SD at person level	σ_*u*_0__	10.60	7.95	12.91	12.39
SD at beep level	σ_*e*_	10.99	10.37	17.91	15.64
**Two-level AR model**	**Notation**	***Neg1***	***Neg2***	***Pos1***	***Pos2***
Avg. trait level	γ_00_	15.54 (1.08)	11.19 (0.90)	57.48 (1.35)	59.01 (1.42)
Avg. inertia beep	γ_10_	0.33 (0.02)	0.33 (0.02)	0.35 (0.02)	0.39 (0.02)
SD at person level	σ_*u*_0__	10.45	7.92	12.94	12.49
SD of inertia beep	σ_*u*_1__	0.18	0.14	0.15	0.14
Residual SD at beep level	σ_*e*_	9.72	9.38	16.52	14.11
Corr. trait level and inertia beep	*r*(*u*_0_, *u*_1_)	0.52	0.53	−0.38	−0.50

*The standard errors for the fixed effects are given between parentheses*.

#### 1.4.2. Two-level AR(1) model

Now we turn to the two-level AR(1) model, in which each affect score is regressed on the immediately preceding affect score of that person. We call the autoregressive coefficient in this model *inertia*, and it reflects the extent to which the person's affect carries over from one moment to the next.

The two-level AR(1) model can be formulated as

(3) $$\begin{array}{lll} \text{Level }1: & y_{bi}=\mu_{i}+\phi_{i}\left(y_{b-1,i}-\mu_{i}\right)+e_{bi}\; & \; \end{array}$$

(4) $$\begin{array}{lll} \text{Level }2: & \mu_{i}=\gamma_{00}+u_{0i}\; & \; \end{array}$$

(5) $$\begin{array}{ll} \phi_{i}=\gamma_{10}+u_{1i},\; & \; \end{array}$$

or as a single equation

(6) $$\begin{array}{ll} y_{bi}=\gamma_{00}+\gamma_{10}\left(y_{b-1,i}-\mu_{i}\right)+u_{0i}+u_{1i}\left(y_{b-1,i}-\mu_{i}\right)+e_{bi},\; & \; \end{array}$$

where μ_*i*_ again represents the trait level of individual *i*, and *e*_*bi*_ now is the part of the person's deviation from his/her trait level at measurement occasion *b* that cannot be explained by the autoregression. The lagged predictor (*y*_*b*−1, *i*_ − μ_*i*_) is centered around the person's trait level, and the parameter ϕ_*i*_ represents the emotional inertia of person *i*. Values of ϕ_*i*_ closer to one indicate higher inertia, which implies strong carry-over of affect, whereas ϕ_*i*_ values close to zero indicate weak carry-over. Values between zero and minus one are possible, but not expected when studying affective inertia (Hamaker and Grasman, 2014), and values larger than one would imply that the process is not stationary.

The reason that we center the lagged predictor as we do, is that we want to estimate each person's trait level μ_*i*_, as well as the population average γ_00_. If the AR(1) model were specified with an uncentered lagged predictor, then it would have a different intercept at the beep level, namely *a*_*i*_ = μ_*i*_(1 − ϕ_*i*_), which would not represent the person's average. The intercept at the second level would also have a different interpretation, not representing the population average trait level of affect. We prefer the centered model notation as it allows us to put a normal distribution on the person's trait level of affect, and, optionally, to predict this trait level using other person-level variables. This makes substantive sense, whereas the uncentered model formulation would put the normal distribution on the substantively uninteresting intercept *a*_*i*_, which seems rather arbitrary and which has the undesirable implication that the trait level itself is assumed to be non-normally distributed in the population (Hamaker and Grasman, 2014). For these reasons, the centered model notation is preferable, but it has the downsides that we need to have an estimate of each person's mean μ_*i*_ beforehand, and that the estimate of the average (fixed) inertia will have a negative bias, as demonstrated by Hamaker and Grasman (2014).

For this model a total of five parameters need to be estimated: The average trait level γ_00_, the average moment-to-moment inertia γ_10_, the variance at the person level $\sigma_{u_{0}}^{2}$, the variance of the inertia $\sigma_{u_{1}}^{2}$, and lastly the residual variance at the beep-level $\sigma_{e}^{2}$. If we add the correlation between the trait level and the inertia we end up with six parameters. But before we can estimate the model, we need to create a new variable in the dataframe containing the lagged, centered predictor. This involves using some estimate for each person's trait level μ_*i*_ around which their predictor is centered. One option is to use the sample mean (i.e., the observed mean of each person's time series) but here we use the empirical Bayes estimate, which results in slightly less bias and higher coverage rates for the fixed inertia parameter (Hamaker and Grasman, 2014).

The empirical Bayes estimates of the trait levels of the persons can be obtained from the empty two-level model. In fact, we can directly obtain the centered predictor values, by using the function resid() to extract the residuals (*e*_*bi*_) from “twolevel.empty,” and storing these as a new variable “e.bi.” Since there were some missing values on the outcome variable, it is important to use subsetting to skip those rows, so that each extracted residual ends up next to the corresponding datapoint in the ESM dataframe:


ESM$e.bi[!is.na(ESM$na)] <-
resid(twolevel.empty)


Now we have the residual stored next to the corresponding affect score, but for our model we need to lag the predictor. With the code below, we make a new variable which, for each row in the data set, equals the *previous* beep's residual, or NA if the previous beep was missing or was on the previous day. This code uses the slide() function from the package DataCombine (Gandrud, 2014). For the first beep of each day, the lagged predictor is assigned a missing value, because we apply the lagging operation only within the same day, as specified through “GroupVar = DayF.” The reason is that it would make no sense to regress the first observation of a day on the previous day's last observation as if they were just two consecutive beeps on the same day. The night represents a significant interruption of the affective process that should be taken into account. Note that for this procedure it is crucial that the data are still ordered as we explained at the beginning of Section 2. After making the lagged predictor, we save “ESM” as a regular dataframe again, which is needed because the slide() function has the side effect of changing some of the properties of the object.


ESM2 <- slide(ESM, Var = “e.bi”, GroupVar =
“DayF”,
NewVar = “lev1pred”, slideBy = -1)
ESM <− as.data.frame(ESM2)


The resulting lagged predictor, stored as ESM$lev1pred has a missing value for the first beep of each day, as well as for those beeps where the person did not provide data on the preceding measurement occasion. Since missing values on predictors are not allowed, when we estimate the AR(1) model the lmer() function will apply listwise deletion to remove all cases with missing predictors from the analysis. It is important to realize that for this reason, the sample size for the AR(1) model will always be smaller than that for the empty model, with a difference of Np*Nd if there are no missing values, and a larger difference if there are.

We can now fit the two-level AR(1) model of Equations (3–5) by specifying a model equation that has “lev1pred” as a predictor with a random effect over persons:


twolevel.AR <- lmer(Neg ~ 1 + lev1pred +
(1 + lev1pred | PersonF),
data = ESM, REML = FALSE)


The model results are given in the bottom part of Table 1 (recall that the outcomes for the first empirical sample are referred to as Neg1 and Pos1, and those for the second sample as Neg2 and Pos2). We see that the average moment-to-moment inertia is between 0.33 and 0.39 for all four outcome variables, with very small standard errors (rounded to 0.02). Since we activated the lmerTest package before running our analysis, the output includes *p*-values (based on the Satterthwaite approximation also used in SAS Proc Mixed, cf. Kuznetsova et al., 2015) for the *t*-tests of the fixed effects in the model^2^. The fixed inertia was significant for each outcome, with *t*_(98.78)_ = 13.2, *p* < 0.001 for Neg1, *t*_(80.75)_ = 15.57, *p* < 0.001 for Neg2, *t*_(95.58)_ = 16.13, *p* < 0.001 for Pos1, and *t*_(75.41)_ = 19.87, *p* < 0.001 for Pos2. The estimated standard deviation of the inertia over persons was also similar across the variables, ranging between 0.14 and 0.18. To obtain a significance test for this random effect, we can use the rand() function from the lmerTest package on the stored model object, which provides us with a likelihood ratio test:


rand(twolevel.AR)


The inertia variance is significant for each outcome, with $\chi_{\left(2\right)}^{2}=155,p<0.001$ for Neg1, $\chi_{\left(2\right)}^{2}=147$, *p* < 0.001 for Neg2, $\chi_{\left(2\right)}^{2}=54$, *p* < 0.001 for Pos1 and $\chi_{\left(2\right)}^{2}=95.5$, *p* < 0.001 for Pos2. This indicates that there are significant between-person differences in the extent of carry-over of negative affect, as well as that of positive affect.

Interestingly, for negative affect we see that the trait level and inertia are positively correlated in both samples, while for positive affect, the trait level and inertia are negatively correlated in both samples. To test the significance of a correlation between random effects, we cannot use the rand() function, but we can fit a constrained model where the correlation is fixed at zero, which is achieved by explicitly separating the random intercept and the random predictor in the lmer() model equation. Then we can use anova() for a likelihood ratio test:


twolevel.AR.nocor <- lmer(Neg ~ 1 +
lev1pred + (1 | PersonF) +
(0 + lev1pred | PersonF),
data = ESM, REML = F)
anova(twolevel.AR, twolevel.AR.nocor)


The correlation turns out to be significant for each of the variables, with $\chi_{\left(1\right)}^{2}=17.4$, *p* < 0.001 for Neg1, $\chi_{\left(1\right)}^{2}=15.2,$ *p* < 0.001 for Neg2, $\chi_{\left(1\right)}^{2}=6.6$, *p* = 0.01 for Pos1, and $\chi_{\left(1\right)}^{2}=12.3$, *p* < 0.001 for Pos2. We can conclude that for negative affect, a higher trait level is associated with more carry-over, while for positive affect, the opposite holds.

### 1.5. Three-level models

We now propose an alternative, three-level modeling approach. As before, we first consider an empty (intercepts-only) model, which accounts for the fact that the beeps are nested within persons, but in this case also accounts explicitly for the multi-day structure of the data by allowing for variation at the day level. Next, we discuss the three-level AR(1) model, which enables us to investigate affective carry-over from day to day and from beep to beep.

#### 1.5.1. Empty three-level model

By using an empty three-level model, we can partition the variance in the affect scores into variance at the person level (level 3), variance at the day level (level 2), and variance at the beep level (level 1). Let *y*_*bdi*_ be the affect score of person *i* on the *b*^th^ beep of day *d*. The model is then given by:

(7) $$\begin{array}{lll} \text{Level }1: & y_{bdi}=\mu_{di}+e_{bdi}\; & \; \end{array}$$

(8) $$\begin{array}{lll} \text{Level }2: & \mu_{di}=\mu_{i}+r_{0di}\; & \; \end{array}$$

(9) $$\begin{array}{lll} \text{Level }3: & \mu_{i}=\gamma_{000}+u_{00i}\; & \; \end{array}$$

where μ_*di*_ represents the mean of individual *i* on day *d*, such that *e*_*bdi*_ is that person's deviation at beep *b* from his or her day mean; μ_*i*_ represents the person's trait level, such that *r*_0*di*_ is the deviation of the current day mean from this trait level; and finally, γ_000_ represents the grand mean of the population, such that *u*_00*i*_ is person *i*'s deviation from the average. The four parameters that need to be estimated for this model are the grand mean γ_000_, the person-level variance $\sigma_{u_{00}}^{2}$, the day-level variance $\sigma_{r_{0}}^{2}$, and the beep-level variance $\sigma_{e}^{2}$. Again, we can obtain a model expression more similar to the R code that we will use below, by writing out the model as a single equation for the predicted value ŷ_*bdi*_:

(10) $$\begin{array}{ll} {ŷ}_{bdi}=\gamma_{000}+r_{0di}+u_{00i}\; & \; \end{array}$$

The model is specified in lme4 by:


threelevel.empty <- lmer(Neg ~ 1 + (1 |
PersonF/DayF),
data = ESM, REML = FALSE)


and, as before, we obtain the model results using the summary() function. The parameter estimates for the four outcome variables are shown in the top part of Table 2. There appears to be substantial variance at the day level for each outcome, although it is clearly smaller than the amount of variance at the person and beep levels. Using rand(), we find that the day-level variance is significant for all outcomes, with $\chi_{\left(1\right)}^{2}=744$, *p* < 0.001 for Neg1, $\chi_{\left(1\right)}^{2}=1148$, *p* < 0.001 for Neg2, $\chi_{\left(1\right)}^{2}=395$, *p* < 0.001 for Pos1, and $\chi_{\left(1\right)}^{2}=1054$, *p* < 0.001 for Pos2. Thus, it appears in each case that a three-level model should be preferred, because a two-level model fails to account for substantial fluctuations in the daily means of affect.

**Table 2:** **The estimated parameters from the three-level empty model and AR(1) model, for negative affect (***Neg***) and positive affect (***Pos***) in the two samples**.

**Three-level empty model**	**Notation**	***Neg1***	***Neg2***	***Pos1***	***Pos2***
Avg. trait level	γ_000_	15.71 (1.09)	11.26 (0.90)	57.41 (1.34)	58.72 (1.40)
SD at person level	σ_*u*_00__	10.36	7.87	12.52	12.18
SD at day level	σ_*r*_0__	5.88	5.36	7.87	7.89
SD at beep level	σ_*e*_	9.57	9.01	16.37	13.69
**Three-level AR model**	**Notation**	***Neg1***	***Neg2***	***Pos1***	***Pos2***
Avg. trait level	γ_000_	15.52 (1.11)	11.19 (0.91)	56.84 (1.39)	58.72 (1.43)
Avg. inertia day	γ_010_	0.10 (0.06)	0.18 (0.06)	0.25 (0.07)	0.28 (0.05)
Avg. inertia beep	γ_100_	0.14 (0.02)	0.09 (0.02)	0.18 (0.02)	0.15 (0.02)
SD at person level	σ_*u*_00__	10.50	7.95	13.06	12.50
SD of inertia day	σ_*u*_01__	0.15	0.25	0.22	0.23
SD of inertia beep	σ_*u*_10__	0.14	0.11	0.15	0.11
Residual SD at day level	σ_*r*_0__	5.11	4.69	6.88	7.09
Residual SD at beep level	σ_*e*_	9.22	8.67	15.81	13.14
Corr. trait level and inertia day	*r*(*u*_00_, *u*_01_)	0.54	0.24	−0.38	−0.52
Corr. trait level and inertia beep	*r*(*u*_00_, *u*_10_)	0.42	0.43	−0.29	−0.44
Corr. inertia day and beep	*r*(*u*_01_, *u*_10_)	0.51	−0.06	0.04	0.40

*The standard errors for the fixed effects are given between parentheses*.

#### 1.5.2. Three-level AR(1) model

To investigate affect regulation in more depth, and in a way that takes into account the three-level structure of the data, we propose using a three-level AR(1) model, in which there are distinct inertia parameters for the carry-over from day to day and from moment to moment. Such a model can be specified as follows:

(11) $$\begin{array}{lll} \text{Level }1: & y_{bdi}=\mu_{di}+\zeta_{i}\left(y_{b-1,di}-\mu_{di}\right)+e_{bdi}\; & \; \end{array}$$

(12) $$\begin{array}{lll} \text{Level }2: & \mu_{di}=\mu_{i}+\beta_{i}\left(\mu_{d-1,i}-\mu_{i}\right)+r_{0di}\; & \; \end{array}$$

(13) $$\begin{array}{lll} \text{Level }3: & \mu_{i}=\gamma_{000}+u_{00i}\; & \; \end{array}$$

(14) $$\begin{array}{ll} \beta_{i}=\gamma_{010}+u_{01i}\; & \; \end{array}$$

(15) $$\begin{array}{ll} \zeta_{i}=\gamma_{100}+u_{10i},\; & \; \end{array}$$

or in the single equation

(16) $$\begin{array}{ll} \;y_{bdi}=\gamma_{000}+\gamma_{010}\left(\mu_{d-1,i}-\mu_{i}\right)+\gamma_{100}\left(y_{b-1,di}-\mu_{di}\right)+ & \;\\ r_{0di}+u_{00i}+u_{01i}\left(\mu_{d-1,i}-\mu_{i}\right)+u_{10i}\left(y_{b-1,di}-\mu_{di}\right)+e_{bdi} & \; \end{array}$$

where μ_*di*_ again represents the mean of individual *i* on day *d*, and *e*_*bdi*_ now is that part of the person's deviation from this day mean at beep *b* that cannot be predicted from their previous centered affect score. Likewise, *r*_0*di*_ represents the part of the day mean's deviation from the person's stable trait level that cannot be explained by the autoregressive relationship, that is, by the regression on the previous day's centered mean. The eight parameters that need to be estimated for the three-level AR(1) model are the grand mean γ_000_, the average day-level inertia γ_010_, the average beep-level inertia γ_100_, the variance across persons of the trait level $\sigma_{u_{00}}^{2}$, of the day-level inertia $\sigma_{u_{01}}^{2}$, and of the beep-level inertia $\sigma_{u_{10}}^{2}$, and the residual variances at the day level $\sigma_{r_{0}}^{2}$ and on the beep level $\sigma_{e}^{2}$. Allowing for all possible parameter correlations gives us a total of eleven model parameters, as there are three parameters that vary across persons (the trait level, the day-level inertia, and the beep-level inertia).

It is important to note that the lagged predictor at the beep level is not the same here as in the two-level AR(1) model of Equations (3–6): Here the predictor is centered around the person's mean for a given day (μ_*di*_), whereas in the two-level model it is centered around the person's trait level (μ_*i*_). The reason for centering the predictor around the day mean is that this allows us to estimate the day mean in Equation (11), instead of an intercept, which in turn allows us to predict this day mean using the autoregressive equation in Equation (12), so that we can look at day to day inertia.

The three-level AR(1) model requires us to create two lagged variables, that is, a within-day centered lagged predictor at the beep level, and a within-person centered lagged predictor at the day level. To start with the first, we can obtain (*y*_*b*−1, *di*_−μ_*di*_) by extracting and then lagging the estimated values of *e*_*bdi*_ from the empty three-level model:


ESM$e.bdi[!is.na(ESM$Neg)] <-
resid(threelevel.empty)
ESM2 <- slide(ESM, Var = “e.bdi”, GroupVar
= “DayF”,
NewVar = “lev1predfor3l”, slideBy = -1)
ESM <− as.data.frame(ESM2)


For the second lagged predictor (μ_*d*−1, *i*_−μ_*i*_) we first need to extract the values of the day-level residuals *r*_0*di*_ from the empty three-level model. The “ESM” dataframe has one line per measurement, and the day-level residual should, by definition, be the same for all measurements on the same day, meaning that it is the same for multiple lines in the dataframe. We can use the ranef() function to extract the day-level residuals from the model object “threelevel.empty” by using the subsetting “$DayF,” but the problem is that missing cases were removed during estimation, so there are fewer residual values than there are lines in our dataframe. To ensure that the residuals are stored alongside the correct datapoints in the dataframe, we first make a vector that contains one entry for each unique “DayF” value. Then we use a for-loop to go through the different day labels, each time extracting the day-level residual for that day (by taking the first of the residuals for that day, which equals all other values in the vector) and assigning it to all the corresponding rows in the dataframe:


di.labels <- unique(ESM$DayF)
for (di in di.labels) {
ESM$r.0di[ESM$DayF==di] <-
ranef(threelevel.empty)$DayF[di,1]}


Having done this, we still need to lag the variable (i.e., shift it by one day) to obtain our lagged predictor. The resulting predictor variable should have missing values (*NA*) for each person's first day, and the days from different persons in the study should never be mixed up. Therefore, we use the code below to do the lagging operation manually, and for each person separately. The first for-loop ensures that we consider each person one by one, and the nested for-loop is used to go through that person's days one by one, with the exception of their first day. The predictor is set to *NA* for each person's first day; for all their later days, the predictor is set to equal the stored residual *r*_0*di*_ of the previous day.


i.labels <- unique(ESM$PersonF)
for (i in i.labels) {
d.labels <- unique(ESM$Day[ESM$PersonF ==
i])
ESM$lev2pred[(ESM$PersonF == i) & (ESM$Day
== 1)] <- NA
for (d in d.labels[-1]) {
ESM$lev2pred[(ESM$PersonF == i) & (ESM$Day
== d)] <-
ESM$r.0di[(ESM$PersonF == i) & (ESM$Day ==
(d-1))][1] } }


Having obtained the necessary lagged predictors, we can proceed by estimating the three-level AR(1) model, allowing for correlations between the random effects:


threelevel.AR <- lmer(Neg ~ 1 + lev2pred +
lev1predfor3l +
(1 | PersonF/DayF) + (1 + lev2pred +
lev1predfor3l | PersonF),
data = ESM, REML = FALSE)


Note that this model will use fewer observations than the empty three-level model, because listwise deletion is applied to remove all cases with missing values on any predictors. Furthermore, since this model has two predictors, and cases are removed whenever at least one predictor is missing, there are also necessarily fewer cases available than for the two-level AR(1) model, which had only one predictor. The reason is that the lagged day-level predictor is, by definition, missing for the first day of each person (as well as in the possible case where a person has missing scores on the measurements for the entire previous day of the study).

As before, the summary() function is used to obtain the model results, which are shown in the lower part of Table 2. For Neg1, the fixed day-level inertia was not significant [γ_010_ = 0.10, *t*_(50.7)_ = 1.7, *p* = 0.09], and the random effect for this inertia was not significant either [σ(*u*_01*i*_) = 0.15, $\chi_{\left(3\right)}^{2}=4.6$, *p* = 0.2]. However, after we removed the non-significant random effect from the model, the fixed effect estimate became larger and significant [γ_010_ = 0.20, *t*_(449)_ = 3.6, *p* < 0.001]. The fixed beep-level inertia and its random effect changed very little between the models and were significant in either case [results from the full model: γ_100_ = 0.14, *t*_(90.4)_ = 5.8, *p* < 0.001; σ(*u*_10*i*_) = 0.15, $\chi_{\left(2\right)}^{2}=38.4$, *p* < 0.001]. The estimates for Neg2 are quite similar to those for Neg1, but one difference is that for Neg2 the fixed and random effect for the day-to-day inertia were both significant [γ_010_ = 0.18, *t*_(64.0)_ = 3.3, *p* = 0.002; σ(*u*_01*i*_) = 0.25, $\chi_{\left(3\right)}^{2}=16.3$, *p* = 0.001]. As in the other sample, the fixed and random effect for the beep-level inertia were both significant [γ_100_ = 0.09, *t*_(68.1)_ = 5.0, *p* < 0.001; σ(*u*_10*i*_) = 0.11, $\chi_{\left(3\right)}^{2}=38.9$, *p* < 0.001].

For positive affect, the fixed day-level inertia was significant in both samples [γ_010_ = 0.25, *t*_(83.9)_ = 3.5, *p* < 0.001 for Pos1, γ_010_ = 0.28, *t*_(70.8)_ = 5.5, *p* < 0.001 for Pos2]. Similar to our findings for negative affect, the individual differences in day-level inertia were not significant for the first sample [σ(*u*_01*i*_) = 0.22, $\chi_{\left(3\right)}^{2}=2.3$, *p* = 0.5], but they were significant for the second [σ(*u*_01*i*_) = 0.23, $\chi_{\left(3\right)}^{2}=10.7$, *p* = 0.01]. Note that the size of the variance estimate is almost equal in the two samples, but the number of days was larger in the second sample, which is likely to have led to a higher power for the significance test of this parameter. The fixed beep-level inertia was significant in both samples [γ_100_ = 0.18, *t*_(88.6)_ = 7.9, *p* < 0.001 for Pos1; γ_100_ = 0.15, *t*_(67.9)_ = 8.1, *p* < 0.001 for Pos2], and so was the random effect of beep-level inertia [σ(*u*_10*i*_) = 0.15, $\chi_{\left(2\right)}^{2}=29.3$, *p* < 0.001 for Pos1; σ(*u*_10*i*_) = 0.11, $\chi_{\left(3\right)}^{2}=30.7$, *p* < 0.001 for Pos2].

Given that we found no significant variance in the day-level inertia for Neg1 and Pos1, it makes no sense to use these outcomes to address the research question whether the regulation mechanisms at the different time scales are correlated. However, we did find significant variance in the day-level inertia parameter for Neg2 and Pos2, so these two outcome variables can be used for this purpose. We can compare the model that we have already estimated to a new model that excludes the random effect correlations, so as to conduct a significance test. The following code fits a three-level AR(1) model without random parameter correlations:


threelevel.AR.nocor <- lmer(Neg ~ 1
+lev2pred +lev1predfor3l +
(1 | PersonF/DayF) + (1 | PersonF) + (0 +
lev2pred | PersonF)+
(0 + lev1predfor3l | PersonF),data = ESM,
REML = F)


The results of the LR test (using the anova() function again) show that the correlations did not improve the model significantly in the case of Neg2, with $\chi_{\left(3\right)}^{2}=7.7$, *p* = 0.05, and we note that the estimated correlation between the two inertias for this outcome was only −0.06, so it appears that these parameters represent distinct regulatory mechanisms. In the case of Pos2, the model was significantly improved by the inclusion of the (three) correlations, with $\chi_{\left(3\right)}^{2}=10.7,p=0.01$. For this outcome, the inertia parameters were both negatively correlated with the trait level [*r*(*u*_00_, *u*_01_) = −0.52, *r*(*u*_00_, *u*_10_) = −0.44], indicating that persons who experience more intense positive affect, on average, tend to have less carry-over from beep to beep and from day to day than persons with a lower trait level. The moderate positive correlation between the two inertia parameters [*r*(*u*_01_, *u*_10_) = 0.44] indicates that higher carry-over between days is associated with higher carry-over from moment to moment.

When comparing the three-level AR(1) model to the two-level AR(1) model, what stands out for each of the four variables is that the average beep-level inertia in the three-level AR(1) model is estimated as substantially smaller (at between 0.09 and 0.18) than the average beep-level inertia from the corresponding two-level AR(1) model (where it ranged from 0.33 to 0.39). Apparently, separating out the day-level variation, as we do in the three-level model by centering the beep-level predictor around the day means, results in a smaller inertia at the beep level. Thus, it appears that there is substantial variation at the day level, and we may be tempted to conclude that the three-level model is more realistic than the two-level model which fails to account for this variation. Furthermore, our results seem to indicate that some of the carry-over of affect actually takes place from day to day (i.e., there is also day-level inertia). However, we cannot decide on this basis that the three-level model is the better model. As we will show in the next section, there is a risk of analytical artifacts or false positives, and distinguishing between two- and three-level processes is less straightforward than it seems.

## 2. Analytical artifacts: confusing day-level variance and beep-level inertia

In the previous section we saw that for each of four empirical outcomes, there was significant variance at the day level and carry-over of affect from day to day. Thus, if we go with the three-level model, based on the variance test, we would conclude that the carry-over of affect from moment to moment may be somewhat smaller than suggested by previous studies using two-level models, because a *part* of the carry-over of affect should properly be understood as taking place from day to day, rather than from moment to moment. However, as we will demonstrate below, distinguishing between a two-level and a three-level model is tricky, because beep-level inertia and day-level fluctuations are hard to distinguish. In this section, we will show that the appearance of significant variance at the day level, and even of significant day-level inertia, does *not* justify the conclusion that the three-level model is more appropriate than the two-level model: The significant day-level variance and day-level inertia can be artifacts caused by the presence of beep-level inertia in the actual two-level model underlying a data set. And in the opposite case, when the three-level model is the actual data-generating model but a two-level AR(1) model is estimated, we run the risk of overestimating the size and significance of the beep-level inertia as a consequence of ignoring the day-level variance.

To demonstrate these problems, we explore the two scenarios by means of fictitious examples. First, we generate data from the empty three-level model of Equations (7–9), which means that there is no inertia at any level, but there *is* variance at each of the three levels. Second, we generate data from the two-level AR(1) model from Equations (3–5), but with measurements spread out over multiple days, as was the case in our empirical examples.

### 2.1. Overestimating moment-to-moment inertia: fitting a 2-level model to 3-level data

To show that variation at the day level may be mistaken for moment-to-moment carry-over in a two-level AR(1) model, we first simulate a data set (*A*) of 90 persons, 21 days, and 10 beeps per day, generated by the empty three-level model given in Equations (7–9). As parameters we used approximately the average over the parameter estimates from the empty three-level model for the two empirical variables Pos1 and Pos2, given in Table 2 (more specifically, we used γ_000_ = 58, σ_*u*_00__ = 12.3, σ_*r*_0__ = 7.9, and σ_*e*_ = 15).

When we fit the two-level AR(1) model from Equations (3–5) to this data (*A*), we find a significant fixed effect for beep-level inertia, with γ_10_ = 0.21, *t*_(95.8)_ = 22.2, and *p* < 0.001. Note, however, that the data were generated without any inertia parameters. The random variance of the inertia is significant too, with σ(*u*_1*i*_) = 0.05, $\chi_{\left(2\right)}^{2}=12.4$, and *p* = 0.002. In comparison, when we fit a three-level AR(1) model, the random effects for the two inertias are nearly zero and are not significant [σ_*u*_01__ = 0.03, $\chi_{\left(3\right)}^{2}=0.60$, *p* = 0.9; σ_*u*_10__ = 0.01, $\chi_{\left(3\right)}^{2}=0.95$, *p* = 0.8]. We remove these non-significant random effects from the model before interpreting the fixed effects. The fixed day-level inertia estimate is not significant [γ_010_ = −0.05, *t*_(1687)_ = −1.5, *p* = 0.13], but the fixed beep-level inertia estimate is significant with γ_100_ = −0.09, *t*_(14660)_ = −10.5, *p* < 0.001. This small but statistically significant negative inertia indicates negative carry-over of affect from moment to moment, which is theoretically not very plausible. A possible explanation is that, as we mentioned above, inertia estimates are negatively biased in a centered AR model (Hamaker and Grasman, 2014). In any case, the estimate is at least *closer* to the true value (zero) than the estimate from the two-level AR(1) model. Note that the residual variances at the three levels are estimated quite accurately with this model.

Repeating the procedure with another simulated data set, *B*, that has a fixed beep-level inertia of 0.15 (without random variance), we find that this parameter is estimated as 0.31 in the two-level AR(1) model, so again the estimates misrepresent the process underlying the data. Moreover, when we create a data set *C* with the same beep-level inertia but with a larger day-level variance (15, which is equal to the beep-level variance, instead of 7.9 as above), the inflation is even more dramatic, with the beep-level inertia now being estimated as 0.55. The size of the population variance at the day level is directly related to how strongly the beep-level inertia estimate in the two-level AR(1) model misrepresents (inflates) the actual moment to moment carry-over of affect.

**Figure 1:**
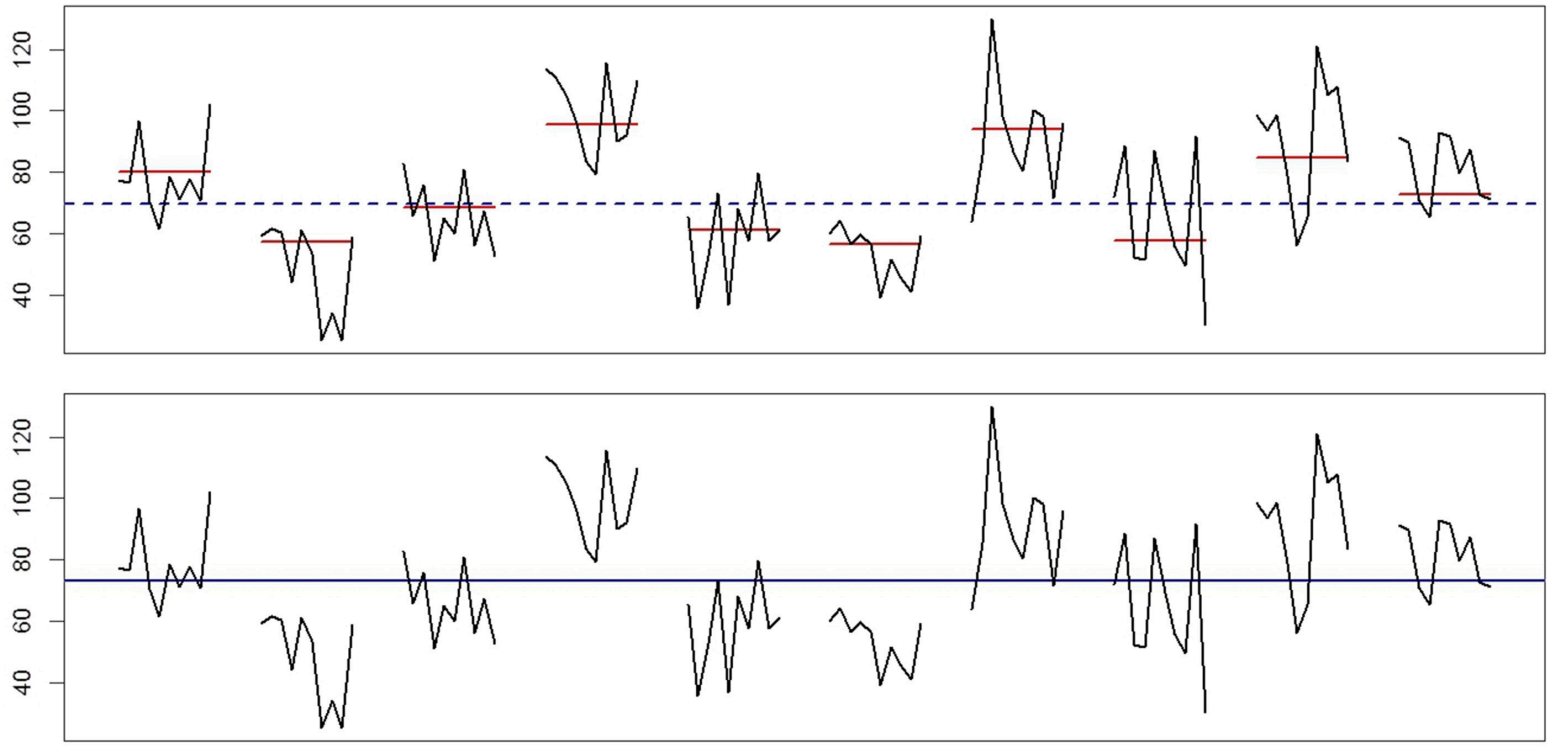
**Example data for one person, generated by a three-level empty model, with randomly varying day means and randomly varying affect scores within days**. The top part shows that relative to the day means (solid lines) there is no visual indication of large carry-over from moment to moment. While inertia is often hard to detect by looking at the data, lack of any visible carry-over does indicate that the inertia, if present, is relatively small, whereas visible carry-over in the data would imply a large inertia. In the bottom part of the figure, where the affect scores are only evaluated against the estimated trait level in the two-level AR(1) model, it appears like there is much carry-over, because entire days are characterized by above-average or below-average scores. Thus, it is no surprise that this model results in a significant estimated beep-level inertia.

To explain why the presence of day-level variance can be mistaken for evidence of (larger than actual) beep-level inertia, we use the time series plot in Figure 1. This is the data for one “person” from another data set, *D*, which was generated by an empty three-level model with the day-level variance and beep-level variance both set at 15 to magnify the effect. The top part of the figure represents the true model, depicting the affect scores together with the person's stable trait level (the dashed line) and the true means for each day (the solid lines). Although we should emphasize that inertia often cannot be detected visually, especially when the inertia parameter is small, our point here is that when the affect scores are evaluated against the means of each day, we see very little indication of carry-over, so our best guess would be that there is little or no beep-level inertia and in this particular case, that would be correct. In contrast, the bottom part of Figure 1 illustrates what happens when we fit a two-level AR(1) model, which means that we ignore the variance at the day level completely and only evaluate the affect scores against the empirical estimate of the person's trait level (the solid line). When we do that, the similarity between affect scores on the same day, which is plain to see, makes it appear as if there must be a lot of carry-over, so it makes sense that the two-level AR(1) model results in a significant beep-level inertia. To put it differently, some of the day-level variance ends up being confused for positive moment-to-moment carry-over.

To summarize, when there is day-level variance in the population and we use an autoregressive model that fails to take this into account, the beep-level inertia becomes inflated, so that we misrepresent the actual process by overestimating the moment-to-moment carry-over of affect. This inflation can even result in finding significant inertia, and significant interpersonal variation in this inertia, when there is zero inertia in the data generating model. So with regard to the empirical applications, at this point the reader may be inclined to put more stock in the lower inertia estimates obtained from our three-level model, and to think that the estimates from the conventional two-level model give a wrong impression of the regulatory process at work in the data. However, as we will see next, we cannot simply conclude that the three-level AR(1) model provides a better indication of the moment-to-moment carry over, since a three-level model can result in finding spuriously significant day-level variance and an *underestimation* of the moment-to-moment carry-over when it is applied to data generated by a two-level model.

### 2.2. Underestimating moment-to-moment inertia: fitting a 3-level model to 2-level data

To illustrate what can happen in the opposite case, that is, when we fit a three-level model to data originating from a two-level model, we simulate a data set *E* (again with *Np* = 90, *Nd* = 21, and *Nb* = 10 per day) from the two-level AR(1) model given in Equations (3–5), using as parameters approximately the averages over the estimates for the two outcomes Pos1 and Pos2, given in Table 1 [more precisely, we use γ_00_ = 58.25, γ_10_ = 0.37, σ_*u*_0__ = 12.75, σ_*u*_1__ = 0.1, σ_*e*_ = 15.25, *r*(*u*_0_, *u*_1_) = −0.44]. Since we assume that the night interrupts the affective process, we do not generate the data for a person as a single long time series stretching out over the days, which would imply that the first measurement of a day is just as strongly related to the last measurement of the previous day as any two other consecutive measurements are related; instead, to incorporate a more realistic view of the nighttime, we generate the time series within each day separately. In other words, the time series for each day has the same person-specific trait level μ_*i*_ and beep-level inertia ϕ_*i*_, but due to the long interruption of the night, the person's affect in the morning is not predicted by how they felt the night before^3^.

When we fit the correct, two-level AR(1) model to this data, the mean and variance of the beep-level inertia are both recovered well, each only differing from the true population value in the second or third decimal. When we estimate the empty three-level model from Equations (7–9), we get significant variance at the day level, with σ_*r*_0__ = 6.15, $\chi_{\left(1\right)}^{2}=943$, *p* < 0.001, which would seem to indicate that we should use a three-level AR(1) model to account for the day-level variance, rather than using a two-level AR(1) model. Thus, we continue by fitting the three-level AR(1) model. We find that the variance of the day-level inertia is not significant [σ(*u*_01*i*_) = 0.11, $\chi_{\left(1\right)}^{2}=0.7$, *p* = 0.40], so we report the other parameters from the re-estimated model after omitting this random effect. The mean beep-level inertia is significant [γ_100_ = 0.25, *t*_(93.4)_ = 18.9, and *p* < 0.001], but gives the impression that the moment-to-moment carry-over is smaller than we would conclude based on the two-level AR(1) model, where it is estimated as 0.37. In the three-level model, we find significant day-level inertia [γ_010_ = −0.09, *t*_(1640.9)_ = −2.4, *p* = 0.01], while we know that the data was generated in such a way that there is no carry-over from day mean to day mean, and any appearance of daily fluctuations in the data is caused by the beep-level inertia. Since the estimate is negative and there is a small sample size at the day level with which to estimate this inertia, the spurious negative inertia can likely be attributed to the negative bias of inertia parameters in a centered model notation (Hamaker and Grasman, 2014).

In summary, due to the nature of autoregressive processes, it is possible for the three-level empty and AR(1) models to show “spurious” significant day-level variance and even day-level inertia when the data actually reflect a two-level AR(1) process that has only beep-level inertia and variance. This means that we cannot reliably use the estimate or the significance test of the day-level variance in the empty three-level model to decide whether a three-level modeling approach should be preferred over a two-level modeling approach. Furthermore, if we use the three-level AR(1) model for inference in the case where the data reflect a two-level process, we end up underestimating the amount of carry-over from moment to moment, but as we have seen in the previous subsection, the opposite can also occur when we apply the two-level model to three-level data. Therefore, we need a better way of deciding how many levels to include in our model.

### 2.3. Model comparison of the AR models

As we have seen, the two-level and three-level AR(1) models can both lead to misleading conclusions about the amount of carry-over when the number of levels in the data does not match the model's assumption. Furthermore, given that the beep-level inertia in a two-level AR(1) process can cause the appearance of significant day-level variance when three-level models are fitted, testing this variance is not a good method of choosing between the two- and three-level approaches, and neither is comparing empty two- and three-level models. A model selection procedure including the AR(1) models seems like a better alternative, because such an approach immediately takes into account the carry-over in the data that could otherwise be mistaken for day-level variance.

A Likelihood Ratio Test (LRT) cannot be used to directly compare the two- and three-level AR(1) model, because these models are not nested; as we noted in the section on the three-level AR(1) model, the beep-level predictor is centered differently. However, the Akaike Information Criterion (AIC; Akaike, 1974) and Bayesian Information Criterion (BIC; Schwarz, 1978) can be used to compare non-nested models, as long as the same cases are used to estimate each model (a requirement that would also apply to a LRT). We will now use the AIC and BIC to choose models for the simulated data sets A to E from above.

The inclusion of the lagged day-level predictor in the three-level AR(1) model causes (more) cases to be removed from the analysis (all cases for which the lagged predictor is missing), so the sample sizes of the two AR(1) models necessarily differ. Therefore, for the purpose of model selection we re-estimate the empty models and the two-level AR(1) model using the smallest subset of the data, that is, those cases that can be used in the three-level AR(1) model. For the purpose of centering the predictors in the AR(1) models we can still use the residuals obtained from the empty models that were fitted to all cases. In this way, we use all the available information to obtain the best possible estimate of each person's trait level and of the day means, around which the predictors are centered in the AR(1) models.

Table 3 contains the AICs and BICs for the simulated three-level data sets *A* to *D* and the two-level data set *E*. In the AR(1) model specifications, we did not include correlations between the random effects, as these were not of interest to us here and would cause a larger difference in complexity between the two-level AR(1) model, in which there can be only one such correlation, and the three-level AR(1) model, in which there can be three. As can be seen in Table 3, for all three outcome variables the AIC and BIC lead us to select a model with the appropriate number of *levels*. We do not always end up precisely with the model that generated the data; outcomes *A* and *D* were generated by an empty model, but the AIC and BIC both favor the AR(1) model. Conversely, outcome *C* was generated by an AR(1) model, but based on the BIC we would select the empty model. However, our purpose here was determining the number of levels, and for this purpose the AIC and BIC both performed flawlessly. Note that it is crucial to include the AR(1) models in the comparison: Comparing only the empty models would lead us to select a three-level model in all cases, including for data set *E*, which was generated by a two-level model. Selecting between the AR(1) models, however, yields the correct number of levels for each of these data sets.

**Table 3:** **Model selection criteria obtained for the simulated data sets A to E**.

** Fit information**		***A***	***B***	***C***	***D***	***E***
AIC	2-l. empty	138151.1	137962.6	145156.6	145250.2	137364.1
	2-l. AR(1)	137420.7	136357.8	139026.2	140601.8	**134641.3**
	3-l. empty	136401.0	136182.0	137790.1	138077.5	136480.8
	3-l. AR(1)	**136299.0**	**136138.1**	**137784.7**	**137935.9**	135445.3
BIC	2-l. empty	138174.2	137985.7	145179.7	145273.3	137387.2
	2-l. AR(1)	137459.2	136396.2	139064.6	140640.2	**134679.8**
	3-l. empty	136431.8	136212.8	**137820.9**	138108.3	136511.5
	3-l. AR(1)	**136360.5**	**136199.6**	137846.2	**137997.4**	135506.8

*The models are estimated on the smallest suitable subset of the data to ensure equal sample sizes. The AR(1) models include the trait level and inertia(s) as random effects, but do not include their correlations*.

*The values in bold indicate which model is selected for that data set*.

### 2.4. Preliminary conclusion

As we showed above, distinguishing between two-level and three-level time series data is tricky because beep-level inertia and day-level variance can easily be confounded. Therefore, a significance test on the day-level variance is not a suitable method of determining whether the model should include this day level in addition to the person and beep levels. Comparing the empty models is similarly problematic because such a procedure does not take into account the effect of possible beep-level carry-over in the data, appearing as spurious day-level variance. Based on our illustration data, using the AIC and/or BIC to select between the AR models seems to be a feasible alternative. Since these results only provided a tentative conclusion, we perform a small simulation study to see whether this approach performs well enough on average to recommend it.

## 3. Simulations

We use simulated data to investigate how well we can distinguish between two-level and three-level AR(1) processes by selecting models based on the AIC or BIC. Given that a three-level data structure is more complex, having day-level variance that is absent in the two-level model, the problem can be formulated in terms of the power to detect a three-level structure, on the one hand, and the risk of false positives (that is, of overfitting) on the other. We investigate the power of the model selection procedure, defined as the proportion of cases where a three-level model is chosen for three-level data, and the Type I error (i.e., overfitting) rate, given by the proportion of cases where a three-level model is selected for two-level data.

### 3.1. Data generation

In generating the data, we used various combinations of sample sizes at each level to investigate how the power of the procedure depends on the design of the study. The number of persons was 30, 60, or 90; the number of days per person was 5, 7, 10, or 14; and the number of beeps per day was 5, 7, 9, or 11. Some of these values are small, but they reflect sample sizes that are realistic in practice, so it is relevant to know whether the procedure would be feasible in these cases. Combining the three factors gave us 48 sample size conditions, and 1000 samples for each condition were generated from a two-level AR(1) model and a three-level AR(1) model. In generating the time series for each person, each day's first measurement was assumed to be independent from the previous day's last, just as in the illustration data in the previous section. All data sets were created without any missing values.

The parameter values for the models were roughly based on the empirical estimates that we obtained for positive affect in the two data sets. More specifically, for the two-level AR(1) data we used γ_00_ = 58, γ_10_ = 0.37, σ_*u*_0__ = 12, σ_*u*_1__ = 0.14, σ_*e*_ = 15 and *r*(*u*_0_, *u*_1_) = −.44. For the three-level AR(1) data the parameter values were γ_000_ = 58, γ_010_ = 0.27, γ_100_ = 0.16, σ_*u*_00__ = 13, σ_*u*_01__ = 0.22, σ_*u*_10__ = 0.13, σ_*r*_ = 7, σ_*e*_ = 15, *r*(*u*_00_, *u*_01_) = −0.45, *r*(*u*_00_, *u*_10_) = −0.365, and *r*(*u*_01_, *u*_10_) = 0.22. In the rare case that a sampled individual inertia parameter (ϕ_*i*_, ζ_*i*_ or β_*i*_) was 1 or larger, it was changed to 0.99 because an inertia of 1 or larger violates the stationarity assumption of an AR(1) model.

### 3.2. Analysis

The first step of the analysis was fitting the empty models to each complete sample to obtain the best possible estimates of all persons' trait levels and centered day means. The residuals from these models were stored to be used as the predictors in the AR(1) models.

Next, for each sample we estimated the two-level AR(1) model from Equations (3–5), omitting random parameter correlations. This model was fitted to the smallest usable subset of the data, that is, to those cases that can also be used to fit the full three-level AR(1) model. Using those same cases, we then fitted several variations on the three-level AR(1) model from Equations (11–15), always omitting random parameter correlations. In the first model variation, a simplification was made by omitting entirely the day-level inertia, in other words, setting the fixed and random effect of day inertia in Equation (14) equal to zero. This means that when we compare it to a two-level AR(1) model, the only difference between the two models is that the three-level model includes day-level variance and has the beep-level predictor centered around the day means instead of around the person's trait level. The second model had a fixed effect for day-level inertia, and the third model added to this a random effect. We compared the AICs and BICs for all the estimated models to see whether the lowest value was obtained for the two-level model or for a three-level model. This way, we focused on the power and the Type I error rate of the decision procedure to choose between two-level and three-level modeling, without making a priori assumptions about specific parameters in the three-level model. Since comparison of the empty models is sensitive to the same overfitting problem as the direct test of the day-level variance, these models were not included in the selection procedure.

### 3.3. Results

The results of the model selection procedure for our simulated samples are presented in Tables 4, 5. From the power values in Table 4 we can conclude that adequate power was obtained in all cases when there were 11 measurements per day, and in many cases when there were 9 measurements per day. It seems clear from our results that the ability to detect day-level variance depends crucially on having at least 9 or preferably 10 measurements per day, regardless of the sample size at the other levels, that is, regardless of the number of participants or days in the study. When the number of beeps per day was lower than this, the power to detect the three-level structure was almost always inadequate given the parameter values used here, and increasing the sample size at the day level then only worsened the problem. This latter result may seem surprising, but makes sense when we consider that the day means are estimated poorly when so few measurements per day are available. It is clear that the number of beeps per day cannot be traded against, or compensated by, the number of days or persons in the study; it is crucial to always have sufficient measurements per day. Another finding is that model selection based on the AIC always provided us with higher power to detect a three-level structure than model selection based on the BIC. This power did not come at the cost of unacceptably higher Type I error rates, because the error rates were always well below .05 for both the AIC and BIC, as can be seen in Table 5.

**Table 4:** **Power to detect the three-level structure for each sample size**.

**AIC**		***Np** = **30***				***Np** = **60***				***N** = **90***			
**Nd**	**Nb:**	**5**	**7**	**9**	**11**	**5**	**7**	**9**	**11**	**5**	**7**	**9**	**11**
5		0.586	0.585	0.700	0.830	0.642	0.685	0.811	0.921	0.700	0.756	0.881	0.954
7		0.390	0.526	0.638	0.821	0.387	0.530	0.738	0.918	0.375	0.530	0.778	0.964
10		0.249	0.440	0.632	0.856	0.198	0.336	0.713	0.934	0.154	0.385	0.768	0.981
14		0.164	0.327	0.632	0.898	0.086	0.273	0.702	0.967	0.046	0.244	0.758	0.986
**BIC**		***Np** = **30***				***Np** = **60***				***N** = **90***			
**Nd**	**Nb:**	**5**	**7**	**9**	**11**	**5**	**7**	**9**	**11**	**5**	**7**	**9**	**11**
5		0.377	0.394	0.529	0.711	0.452	0.522	0.674	0.851	0.527	0.594	0.777	0.910
7		0.252	0.391	0.525	0.746	0.261	0.421	0.663	0.883	0.263	0.443	0.710	0.935
10		0.171	0.350	0.540	0.793	0.131	0.268	0.640	0.910	0.115	0.299	0.695	0.962
14		0.107	0.231	0.545	0.846	0.056	0.216	0.619	0.952	0.027	0.185	0.696	0.972

*The power was defined as the proportion of three-level data sets for which one of the three-level models was selected*.

**Table 5:** **Type I error rates for each sample size**.

**AIC**		***Np** = **30***				***Np** = **60***				***N** = **90***			
**Nd**	**Nb:**	**5**	**7**	**9**	**11**	**5**	**7**	**9**	**11**	**5**	**7**	**9**	**11**
5		0.048	0.008	0.000	0.000	0.008	0.000	0.000	0.000	0.005	0.000	0.000	0.000
7		0.004	0.000	0.000	0.000	0.000	0.000	0.000	0.000	0.000	0.000	0.000	0.000
10		0.000	0.000	0.000	0.000	0.000	0.000	0.000	0.000	0.000	0.000	0.000	0.000
14		0.000	0.000	0.000	0.000	0.000	0.000	0.000	0.000	0.000	0.000	0.000	0.000
**BIC**		***Np** = **30***				***Np** = **60***				***N** = **90***			
**Nd**	**Nb:**	**5**	**7**	**9**	**11**	**5**	**7**	**9**	**11**	**5**	**7**	**9**	**11**
5		0.007	0.001	0.000	0.000	0.002	0.000	0.000	0.000	0.000	0.000	0.000	0.000
7		0.000	0.000	0.000	0.000	0.000	0.000	0.000	0.000	0.000	0.000	0.000	0.000
10		0.000	0.000	0.000	0.000	0.000	0.000	0.000	0.000	0.000	0.000	0.000	0.000
14		0.000	0.000	0.000	0.000	0.000	0.000	0.000	0.000	0.000	0.000	0.000	0.000

*The Type I Error rate was defined as the proportion of two-level data sets for which one of the three-level models was selected*.

### 3.4. Conclusion

Based on our simulations we can conclude that model comparison of the AR models, using the AIC or BIC, is a suitable method of deciding whether to specify two or three levels. The AIC is to be preferred over the BIC, as it results in a higher power while the Type I error risk remains low. Overall, it was clear that the number of measurements per day should be around ten or higher, regardless of the sample size at the other levels, for adequate power. This is not really surprising given that even the most stripped-down version of the three-level model involves estimating a mean for each separate day. When our simulated data had a sufficient number of measurements per day, the power was adequate even for the smallest numbers of persons and days and varied only little depending on these sample sizes. We used empirically derived parameter values for the simulated data, with some inertia present both at the beep level and at the day level. Although, we did not investigate with simulations the exact performance of this model selection procedure when different parameter values apply, our results indicate that model comparison of the AR models using the AIC is a viable approach to deciding on the number of levels, whereas inspecting or testing the day-level variance in the empty models are not viable approaches in this context.

**Figure 2:**
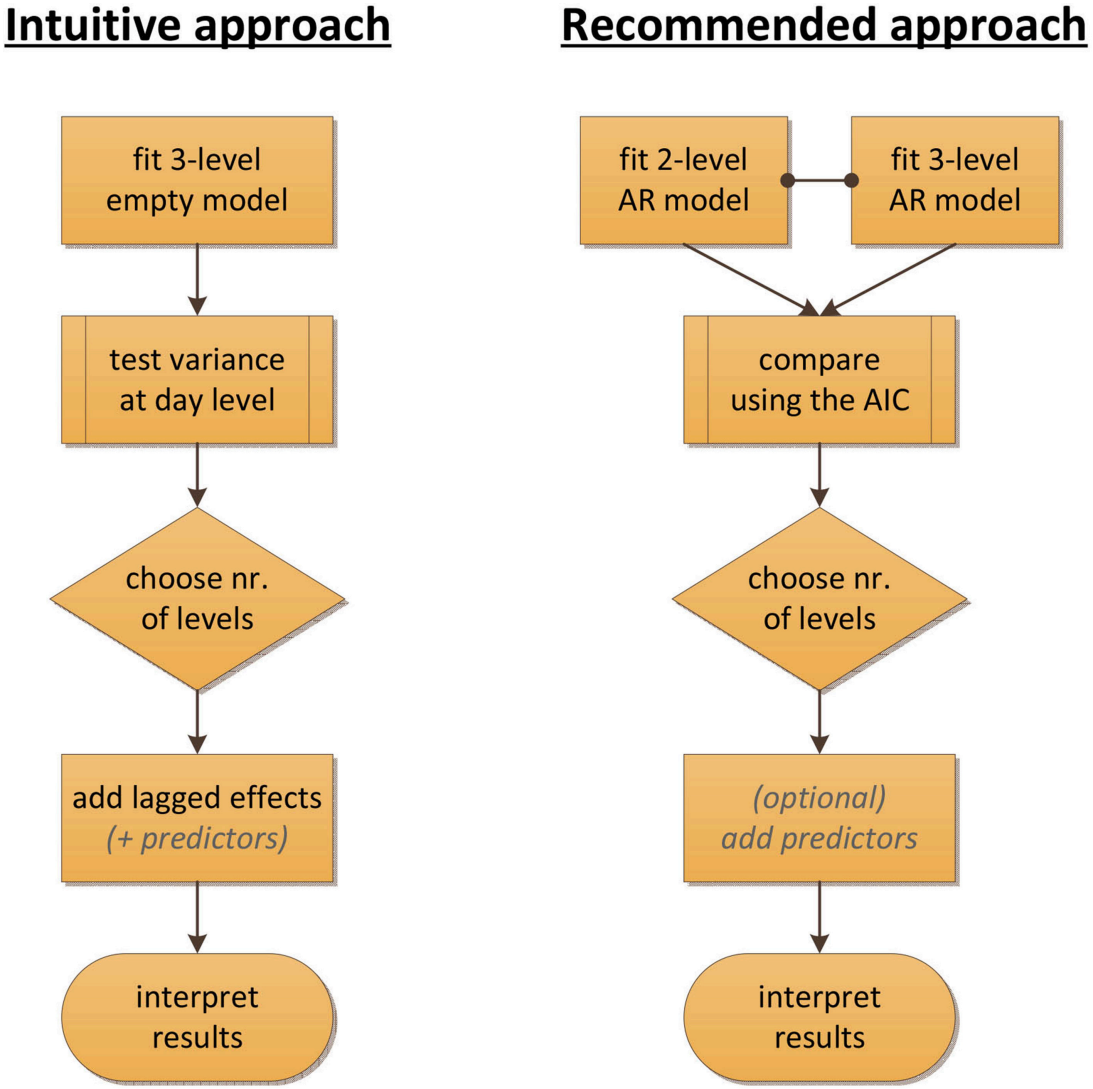
**Flowcharts contrasting the intuitive and the recommended approach for determining the number of levels in the context of time series modeling of ESM data**. The intuitive approach would be to inspect or test the variance at the day level first, using empty models. However, such an approach breaks down when there is carry-over in the data. The recommended alternative is to start by fitting AR models that account for this carry-over, and to use the AIC to then select between these models.

Figure 2 summarizes our conclusion by contrasting two procedures for deciding on the number of levels. The intuitive procedure, based on common practice in multilevel modeling, would be to test the day-level variance first (either directly, or by a comparison of the empty models) before continuing with the specification of AR models, but in the context of time series data, this approach clearly breaks apart due to the possibility that carry-over is mistaken for day-level variance. The alternative approach that we recommend here is to start by specifying the AR models and then select between these models, using the AIC, to determine the number of levels needed.

## 4. Empirical cases revisited

Having established that the AIC can be used to decide whether to include a day level in the AR model, we revisit our analyses of negative and positive affect in the two empirical samples. In the same way as we did before, the empty models were fitted to the complete data in order to make use of all the available information for centering the lagged predictors around the persons' trait levels and day means. But for the purpose of comparing the AIC of the AR(1) models, the two-level AR(1) models were refitted to the smallest of the data, that is, to those cases that could also be used in the three-level AR(1) model with day-level inertia.

The AICs for all fitted AR(1) models are given in Table 6. For each of the four empirical outcomes, the two-level AR(1) model has the lowest AIC. Although there were some missing values in these empirical outcomes, unlike in our simulated data, the sample sizes at all levels were quite large, so that there is no compelling reason to think that the choice of a two-level model must have been caused by a lack of power to detect day-level variance. The differences in the AICs were very large and clearly indicate that the two-level AR(1) model is more appropriate for these data than the three-level AR(1) model, so we can conclude that our analysis does not lend support to the notion that there are daily fluctuations in affect that need to be taken into account.

**Table 6:** **AICs obtained for the four empirical variables, using the same cases as outcomes in each model**.

**AIC values**	**Neg1**	**Neg2**	**Pos1**	**Pos2**
2-level AR(1)	**31771.9**	**50941.2**	**36163.5**	**56649.1**
3-level AR(1) without beta	31973.5	51207.2	36332.8	56953.3
3-level AR(1) with fixed beta	31962.4	51183.3	36317.8	56904.3
3-level AR(1) with random beta	31962.1	51170.6	36318.8	56900.0

*In these AR(1) models, correlations between random parameters were not included*.

*The values in bold indicate which model is selected for that data set*.

Note that the empirical analyses in the current study focused on the issue of how many levels should be specified in the context of the commonly used AR(1) model for affect. We did not conduct exploratory analysis (e.g., inspection of the partial autocorrelation function or the power spectrum for each person's data) or comparisons with alternative or more complex modeling approaches (e.g., higher-order AR models, or models with linear or cyclic trends). As such, our analysis provided an empirical illustration of the relevance of the day-level question for ESM data, but it should not be taken as the definitive analysis of these particular data sets, or as evidence that a 2-level AR(1) model is the best analysis method as a general matter. In the discussion we will consider the broader context of affect dynamics research, as well as some limitations of the AR modeling approach studied here.

## 5. Discussion

The present study was inspired by the observation that two-level AR(1) models have been used in affective research to study inertia at the day-to-day level, e.g., using diary data, as well as inertia at a moment-to-moment level, using ESM data with multiple measurements within a day. Given the findings from both types of research, it seems clear that there is carry-over of affect both within and between days. While ILD frequently contains multiple days and multiple measurements within days, the commonly used two-level modeling approach does not account for daily fluctuations in affect, or for carry-over from one day to the next. In this paper, we proposed a three-level AR(1) model that allows us to investigate the variability and the inertia (carry-over) of affect within and between days simultaneously, and we illustrated how two- and three-level AR models can be implemented correctly for ESM data using R.

Surprisingly, our empirical findings, based on positive and negative affect data from two ESM studies, did not lend support to the idea of carry-over of affect from day to day. In fact, our findings would suggest that there is no relevant variance at the day level, that is, there is no variability in the daily means of affect, except as an artifact caused by the variability from moment to moment. This raises some questions about how to interpret and combine the findings from different studies. Do the measures from diary studies, where people report their affect once a day, reflect something different than an average of their affect throughout the day? How should we interpret the variability and inertia that is found in those data sets with daily measurements? It is known that retrospective reporting can suffer from various types of bias (for examples, see e.g., Fredrickson and Kahneman, 1993; Trull and Ebner-Priemer, 2009), so perhaps once-daily measurements do not always represent the average affect levels of the day accurately. On another note, it may be that an accurate estimation of day means requires more measurements per day than we had in our empirical data.

Apart from these substantive questions, in this paper we have argued that it is a matter of general importance for researchers working with nested ILD to consider the number of levels in their data carefully, and to decide whether or not the day should be included as a level in their model. When autocorrelation in this type of data is not accounted for, researchers may end up using models with more levels than appropriate. At the same time, when there is variance at the day level that is not accounted for, researchers who investigate autocorrelation may end up with misleading results. Thus, our advice is that researchers carefully investigate whether or not they need to include a day level, before interpreting the results of a specific model. We demonstrated why a common approach that makes sense in other contexts—evaluating the variance components in intercept-only (empty) models—is not viable in this context. Based on our simulations we recommend using the AIC to compare AR models with and without a day level, since this approach seems to minimize the risk of overfitting while offering a reasonable power to detect day-level variance. For this procedure to work adequately, it is important to have at least nine, but preferably more measurements per day.

The current study focused on one particular approach in affect dynamics research, namely AR(1) modeling to investigate carry-over or inertia. A well-known limitation of this modeling framework is that it assumes equally spaced measurements, an assumption that is clearly violated in ESM data collection, where the intervals between measurements are deliberately varied. It is not clear how much of a problem this is in practice, since AR models for ESM data have yielded valuable insights into affect dynamics (e.g., Suls et al., 1998; Koval and Kuppens, 2011), and the variability of intervals in an ESM design is modest. While continuous-time extensions of AR (and other) models can be implemented in software like the ctsem package for R (Driver et al., submitted), it is not yet possible to estimate random inertia parameters in that framework. Thus, avoiding the assumption of equally spaced measurements would come at the cost of making a different, more problematic assumption, namely, that there are no individual differences in the carry-over of affect from moment to moment, or alternatively, having to fit a separate model to each person's time series.

To summarize, we hope that researchers who analyze ILD, and especially those who are interested in inertia, will be aware of the potential issues resulting from having measurements nested not only within persons, but within multiple days per person. To gain a better understanding of how inertia within days and between days is related, further research is needed that addresses potential complicating factors such as time trends (Wang and Maxwell, 2015) and the role of recall or other biases in self-reported affect measures (Fredrickson and Kahneman, 1993; Trull and Ebner-Priemer, 2009). Furthermore, it is worth noting that AR modeling is but one among many approaches to investigating affect (cf.Hamaker et al., 2015; also see Houben et al., 2015), and it is still an open question what type of analysis can best capture the complex dynamics of affect in daily life, which may well involve linear or cyclic trends (e.g., Ram et al., 2005), longer-range serial dependence (Wagenmakers et al., 2004), state-switching (Hamaker and Grasman, 2012), or chaotic elements (e.g., Heiby et al., 2003). Integrating the findings from different modeling paradigms as well as from studies measuring affect at different time scales is a major challenge in the quest for a comprehensive understanding of affect dynamics and between-person differences therein.

## Author contributions

SH conducted all analyses and simulations, wrote first drafts of all manuscript sections and incorporated revisions based on the suggestions and feedback from PK and EH. PK contributed the (two) empirical data sets, and critically revised the content of the study and the conducted analyses as well as the style and structure of the manuscript. EH proposed the research question for the study and the methodological approach, and the focus and style of the manuscript; and contributed substantially to the conception and revision of the manuscript and the choice of simulations/analyses.

## Funding

This study was supported by a Grant from the Netherlands Organization for Scientific Research (NWO VIDI 452-10-007) to EH, and Grants GOA/15/003 and OT/11/31 from the Research Fund of KU Leuven, IAP/P7/06 from the Interuniversity Attraction Poles program financed by the Belgian government, and G093512N from the Fund for Scientific Research-Flanders.

### Conflict of interest statement

The authors declare that the research was conducted in the absence of any commercial or financial relationships that could be construed as a potential conflict of interest.

## Footnotes

^1^The notation AR(1) indicates a first-order autoregressive model, i.e., a model where each observation is predicted from the one immediately preceding it. Here we do not consider higher-order autoregressive models.

^2^Other options for significance testing for mixed models fitted with the lme4 package include the use of bootstrapped or profile likelihood confidence intervals, which can be obtained using the confint() function.

^3^If we assume that the affective process continues in exactly the same way during the night as it does during the day, only unobserved, the autoregressive coefficient for predicting the morning measurement from the previous night's last measurement is still effectively zero, because it is given by ϕ raised to a high power.
